# Genetics Talks to Epigenetics? The Interplay Between Sequence Variants and Chromatin Structure

**DOI:** 10.2174/138920210791616662

**Published:** 2010-08

**Authors:** Silvio Zaina, Elva L Pérez-Luque, Gertrud Lund

**Affiliations:** 1 Department of Medical Research, Division of Health Sciences, Leon Campus, University of Guanajuato, Leon, Mexico;; 2 Department of Plant Genetic Engineering, CINVESTAV Irapuato Unit, Irapuato, Mexico

**Keywords:** Chromatin, DNA methylation, epigenetics, genetic variant, histone, single nucleotide polymorphism.

## Abstract

Transcription is regulated by two major mechanisms. On the one hand, changes in DNA sequence are responsible for genetic gene regulation. On the other hand, chromatin structure regulates gene activity at the epigenetic level. Given the fundamental participation of these mechanisms in transcriptional regulation of virtually any gene, they are likely to co-regulate a significant proportion of the genome. The simple concept behind this idea is that a mutation may have a significant impact on local chromatin structure by modifying DNA methylation patterns or histone type recruitment. Yet, the relevance of these interactions is poorly understood. Elucidating how genetic and epigenetic mechanisms co-participate in regulating transcription may assist in some of the unresolved cases of genetic variant-phenotype association. One example is loci that have biologically predictable functions but genotypes that fail to correlate with phenotype, particularly disease outcome. Conversely, a crosstalk between genetics and epigenetics may provide a mechanistic explanation for cases in which a convincing association between phenotype and a genetic variant has been established, but the latter does not lie in a promoter or protein coding sequence. Here, we review recently published data in the field and discuss their implications for genetic variant-phenotype association studies.

## CHALLENGES IN DETERMINING GENETIC VARIANT-PHENOTYPE ASSOCIATIONS

### Sequence Variants Located Outside Promoter and Protein Coding Regions

Genetic variants (GVs) are distributed across the genome in both coding and non-coding sequences. In fact, selective pressure tends to decrease the frequency of GVs within exons, in comparison with promoters and introns. This phenomenon has been clearly illustrated by a study addressing the genome-wide distribution of single nucleotide polymorphisms (SNPs) [1]. The authors found average SNP densities (*i.e.* number of SNPs per 10 kb) of 8.33, 8.44, and 8.09 in the whole genome, in intergenic and genic regions, respectively. A closer look at genic regions revealed SNP densities of 8.21, 5.28, and 7.51 in intronic, exonic and 5’ or 3’ untranslated regions, respectively. The implication of these observations is that a vast number of non-coding sequence GVs is likely to be identified by genetic screenings as potential phenotype modifiers. This conclusion has been supported by a recent meta-analysis of genome-wide genetic studies showing that a large proportion (39%) of SNPs associated with 22 common human diseases are intergenic [2]. Yet, traditional views of gene function and regulation tend to filter out non-coding sequence GVs as functionally neutral, while stressing the importance of promoter or exon GVs in transcription factor binding or reading frame alteration and consequent phenotypic modification. A further complication is that non-coding GVs often lie within genes that represent excellent candidate phenotype modifiers based on their function. The following are representative examples of many found in the literature. One such example is the transcription factor 7-like 2 (*TCF7L2*) gene and the role of SNPs present in that gene in type 2 diabetes. *TCF7L2* encodes a high mobility group box-containing transcription factor that has been implicated in blood glucose homeostasis [3]. Genetic variants of this gene are associated with increased risk of type 2 diabetes, and extensive genome-wide association studies have identified *TCF7L2* as a type 2 diabetes susceptibility gene. Noticeably, *TCF7L2* SNPs have been demonstrated to have by far the biggest effect on the risk of developing type 2 diabetes, in comparison with all SNPs studied to date. In particular, SNPs located in *TCF7L2 *intron 4 and 5 – *i.e.* rs12255372 and rs7903146 – have shown robust associations with type 2 diabetes in a Danish and US cohort [4]. These observations are supported by independent studies in other ethnic groups [5]. How these SNPs located in introns affects the expression of TCF7L2, and therefore the risk of type 2 diabetes, remain largely unknown. A second relevant example is rs9939609, a SNP located in the fat mass and obesity associated (*FTO*) gene. *FTO* has attracted a huge interest, as it was identified by genome-wide studies as a susceptibility gene for type 2 diabetes. Furthermore, diabetes risk-associated *FTO* alleles were also strongly associated with increased body mass index (BMI), a measure of obesity, strongly suggesting that the association of *FTO* GVs with type 2 diabetes risk is secondary to effects on BMI. The relevant GV is in this case a cluster of 10 SNPs in the first intron of *FTO* that are associated with both traits [6]. rs9939609 was used in all further studies because among the cluster of most highly associated SNPs it had the highest genotyping success rate (see for example the recent study by Gu *et al.* [7]). Another useful example is the association between receptor estrogen alpha (*ESR1*) gene GVs and endometrial cancer. ESR1 is the main estrogen receptor expressed in the endometrium and has been proposed to play a pivotal role in determining endometroid endometrial carcinoma risk, the most common histological subtype among endometrial malignancies [8]. Among *ESR1* SNPs analyzed, rs9340799A/G and rs2234693T/C were associated with a significant reduction in disease risk. rs9340799GG genotype associated with nearly 50% decreased risk for endometrial cancer compared to AA genotype (OR 0.53; CI 0.37-0.77), while the effect of rs2234693C/C genotype was less pronounced (OR 0.65 CI 0.48-0.89) [9]. These SNPs are found in a 46 bp region of *ESR1* intron 1.

### Genetic Variants that Lie in Biologically Relevant Genes but are Poorly Associated with Phenotype

Another type of challenge faced at times by genetic association studies is the dilemma of biologically strong candidate genes containing GVs that perform very poorly in genotype/phenotype association tests. A further layer of complexity is that the significance of association tests in some cases varies significantly between studies conducted in different countries and ethnic groups. Again, examples of GVs with these characteristics abound in the literature and involve genes for which a pivotal role in various important diseases has been proposed based on functional data. The Q223R polymorphism in the leptin receptor gene (*LEPR*) is one example. LEPR mediates the main biological effects of leptin, *i.e.* the imposition of a negative feedback signal in regulating body-weight through reduction of food intake and stimulation of energy expenditure [10]. Leptin being a fundamental factor in body weight regulation, the identification of GVs modulating its pathophysiological effects is of potentially enormous importance. The *LEPR* GV in question consists of an A to G transition at position 668 in exon 6, resulting in a substitution of an arginine for glutamine located in the extracellular domain of the encoded receptor protein, thus making this GV a seemingly straightforward predictor of biological function [11]. The results of genetic association studies for *LEPR* Q223R polymorphism vary dramatically across different studies. On the one hand, the observed allelic frequency differs among countries and ethnic groups. In particular, the frequency of the 223R allele for Asians was significantly higher than for other ethnicities [12]. On the other hand, significant effects of this GV on obesity were reported in two early meta-analysis [12, 13], and was confirmed by more recent studies [14-18]. However, its association with obesity is generally regarded as controversial, given that reports showing only weak or no association at all have been published [19, 20].

A second relevant example is the S810L point mutation of the mineralocorticoid receptor (*MR*) gene. This is a TCA to TTA missense mutation at nucleotide 810 in exon 6 that results in substitution of serine with leucine in the hormone-binding domain of the encoded receptor. This change leads to an increased, ligand-independent baseline activity of the receptor and consequent hypertension [21]. In addition, the mutation increases the affinity of MR for progesterone and confers agonist activity to other steroids that are normally MR antagonists. The mutation showed a precise (100%) association with repeated severe pregnancy-induced hypertension in a US population sample [22]. This very convincing result contrasts with the conclusions of a recent work by our group addressing the mutation association with gestational hypertension. The latter study found similar genotype frequencies among hypertensive and normotensive women (12% *vs* 9.4%) [23], thus suggesting that population-specific differences play a significant role in this case. A further complication affecting the study of the *MR* S810L mutation is its extreme rarity or absence in selected populations, as demonstrated by studies including women affected by pregnancy-induced hypertension, preeclampsia and essential hypertension [24-26].

Another factor implicated in metabolic diseases is adiponectin (ADIPOQ), representing the most abundant adipocyte-derived protein. ADIPOQ exhibits antiatherogenic and antiinflammatory properties, in addition to induce antidiabetic effects due to insulin sensitizer activity [27, 28]. In accordance with its biological activities and perhaps in contrast with the fact that ADIPOQ is produced mainly in the adipose tissue [29], obese subjects have significantly lower plasma ADIPOQ concentration than non-obese subjects [30]. Adiponectin levels have a strong genetic component, with heritability estimated between 30 and 50% [31]. Therefore not surprisingly, *ADIPOQ* has been identified as an important susceptibility locus for metabolic syndrome, type 2 diabetes and cardiovascular disease [32, 33]. In particular, the association of 5´ region SNPs rs17300539 (-11391G>A) and rs266729 (-11377C>G), rs2241766 (45T>G) in exon 2 and rs1501299 (276G>T) in intron 2 with adiponectin level, insulin resistance and obesity has been extensively studied, but often with inconsistent results. For instance, the *ADIPOQ *45G allele was associated with higher risk of obesity and insulin resistance in a German population [34] but protective among Taiwanese [35, 36]. Lack of consistency has been observed for *ADIPOQ *276G>T polymorphism in which the increased risk of obesity and insulin resistance was associated with 276T allele among Italians [37] but with 276G allele among Greek women [38]. The SNPs -11391G>A and -11377C>G had shown strong association with adiponectin levels [39-41]. The 45T/G and 276G/T SNPs also have been associated with serum adiponectin [39, 41, 42].

As expected, the inconsistent associations involving the GVs presented above have been the objective of much speculation. Although a number of factors such as sample size could be responsible, it is possible that yet poorly characterized genetic regulatory mechanisms, differential environmental or diet exposure play an additional, perhaps equally important role. In the following paragraphs, we will discuss how epigenetics provides a conceptual framework that takes into account allele-environment and allele-diet interactions and may improve our understanding of complex genotype-phenotype associations.

## EPIGENETICS, CHROMATIN

Although various definitions of epigenetics have been proposed [43], we favour the one that describes epigenetics as the study of mechanisms of gene regulation that are dependent on chromatin architecture. This definition is distinct from the one of functional genetics, which singles out DNA sequence variants as determinants of gene expression. Chromatin is a high-order structure that in addition to serve the purpose of compacting the ~2 m.-long DNA chain contained in a typical high eukaryote cell nucleus, regulates gene transcription. Nuclear chromatin is a nucleoprotein complex in which DNA is wrapped around octamers of histone proteins called “core histones”, which include two copies of each histone type 2A, 2B, 3 and 4. The fundamental repetitive unit of chromatin is called nucleosome, defined as one core histone octamer and the DNA wrapped around it. Transcription of a given gene is affected by the architecture of chromatin in which the gene is embedded. Generally speaking, chromatin architecture can be classified into two main types, a compact one in which genes are silent (non-permissive chromatin), and a more relaxed one, permissive of transcription, in which gene promoters are accessible to the transcription machinery [44].

A number of molecular modifications of DNA and histones constitute epigenetic marks that are specifically associated with permissive or non-permissive chromatin. DNA methylation and histone posttranslational modifications are the most studied epigenetic marks. DNA methylation in mammals occurs mostly at position 5 of cytosine residues in a CpG dinucleotide context to yield 5-methyldeoxycytidine (5mdC). The various known histone modifications include acetylation, methylation, phosphorylation and sumoylation. A specific combination of epigenetic modifications determines or reflects the transcriptional status of a given DNA regions. For example, DNA hypomethylation, histone hyperacetylation and hypomethylation of Lys 20 of histone 4 are modifications generally associated with active DNA regions, whereas the reciprocal modifications are present in inactive regions of the genome [44]. In addition, regulatory element-specific epigenetic marks have been uncovered. For example, promoters and enhancers display characteristic nucleosome-free sites but differ in the degree of histone 3 Lys 4 methylation [45]. Although several exceptions probably exist, epigenetic marks occupy a region of variable size rather than being punctual and when *de novo* imposed often spread to adjacent sequences, as early work on promoter DNA methylation demonstrated [46]. 

All epigenetic modifications present in a given cell nucleus at a given point in time are collectively referred to as epigenome. The epigenome is at least in part a plastic entity, *i.e.* cell growth and differentiation are often associated with changes in DNA methylation status and collection of histone modifications at specific loci. One extreme example of this phenomenon is the widespread erasure and re-establishment of DNA methylation patterns during mammalian embryo development, clearly illustrating the reversible character of epigenetic changes [47]. Epigenetic patterns differ between different cell types, implying that different epigenomes coexist in a genetically uniform organism [48]. Indeed, plasticity is a unique characteristic of the epigenome, which can be described as a fluctuating entity during development and cell differentiation, distinct from dynamics that shape the genome, *i.e.* generally irreversible mutations. Despite being plastic, epigenetic marks are maintained upon cell division, indicating that a degree of stability is another fundamental property of epigenetic marks at least in a homogeneous, not differentiating cell population.

Deviations from the physiological epigenome are believed to play a significant role in numerous diseases such as cancer, mental disorders and atherosclerosis [49, 50]. In the case of cancer, a frequently observed epigenetic change is global loss of 5mdC (DNA hypomethylation) and concomitant hypermethylation of a subset of CpG-rich regions called CpG islands, typically located in promoter regions. Given the occurrence of epigenetic alterations in disease, much effort is directed towards understanding what factors regulate the equilibrium between stability and dynamic changes of the epigenome, and their mechanisms of action. It is clear that factors exogenous to the organism play a role in these changes. Broadly speaking, these exogenous forces include at least diet and environmental factors. Seminal observations in animal models and in humans provide proof of principle that diet and environment act indeed as epigenome modifiers [51, 52]. In particular, specific environmental risk factors for cardiovascular disease have been recently linked to hypomethylation of a substantial part of the genome in humans [52]. Based on these observations, it is widely believed that changes in the population’s exposure to diet-related and life style-related factors induce epigenetic changes that in turn contribute to the recent epidemics of metabolic diseases such as obesity, diabetes, metabolic syndrome and vascular complications [53].

## GENETIC VARIANTS CAN AFFECT EPIGENETIC MARKS

The concepts presented in the last paragraph offer hints to understand on the one hand how non-promoter and non-coding GVs can impact on transcription, and on the other hand why genetic associations are in some cases unexpectedly poor or inconsistent in different studies. In both instances, it is possible that GVs are associated with changes in DNA methylation patterns or histone marks, which in turn can provoke rearrangements of chromatin architecture that may have long-range transcriptional effects. For example, an intronic SNP-imposed DNA methylation state, if affecting the methylation state of a nearby promoter, would in principle exert the same effect as a transcription factor binding site-disrupting mutation. In the next paragraphs, we provide examples of evidence and proposed mechanisms that support the existence of cross-talks between genetic background and epigenetic marks.

### Sequence-Specific Regulation of DNA Methylation

A number of observations indicate that GVs have an impact on DNA methylation. One well-known, straightforward mechanism is the depletion of methylable sites, *i.e.* CpG dinucleotides. Work in the late eighties has demonstrated that G/A (C/T in the opposite strand) transitions are the most common point mutations in the human v-Ki-ras2 Kirsten rat sarcoma viral oncogene homolog (*KRAS*) gene [54]. Genome-wide scale studies subsequently confirmed these early observations [55]. In particular, the latter study generalized previous observations that C/T transition is a major force resulting in the depletion of genome CpG dinucleotides, a phenomenon that is at least in part driven by natural decay of 5mdC. Thus, it can be proposed that GVs can epigenetically regulate transcription by disrupting binding sites for regulatory proteins that recognize 5mdC. Disrupting events can include CpG destruction, as 5mdC-binding factors can bind to sequences containing a single 5mdCpG dinucleotide, or loss of A/T runs close to methylated sites needed by some of those factors for DNA binding [56, 57]. At least two studies demonstrate that punctual loss or gain of DNA methylation secondary to destruction of a single CpG nucleotide by a T/C or G/A SNP, greatly affects promoter activity of matrix metalloproteinase 1 (*MMP1*) and potassium-chloride co-transporter 3 (*SLC12A6*) genes [58, 59]. Noticeably, those SNPs are medically relevant as have been linked with risk of preterm premature rupture of membranes and psychiatric disorders, respectively. Furthermore, if a GV results in punctual depletion of a methylable site and this has spreading effects on adjacent sequences, one would expect to observe an association between DNA methylation state and proximal DNA sequence. This prediction has been demonstrated to hold true by a seminal work showing that specific SNP genotypes associate with specific DNA methylation patterns and possibly act as modifiers of DNA methylation *in cis* [60]. By interrogating SNP arrays with methylation-filtered genomic DNA obtained from a number of human tissues, the authors showed that allele-specific DNA methylation (ASM) was specifically associated with adjacent SNP genotype in a number of loci. Importantly, SNP genotype-specific DNA methylation states were strongly correlated with allele-specific expression. A recent study expanded these observations by addressing the impact of SNPs located at CpG dinucleotides on ASM [61]. The work stemmed from the observation that one characteristic epigenetic feature observed during nuclear reprogramming is the presence of intermediate CpG methylation states – *i.e.* ~50% - at selected loci, even in monoclonal cell lines. Since this intermediate methylation state could not be explained by imprinting or X chromosome inactivation, the authors turned their attention to ASM. The study was conducted in various human pluripotent cell lines and constitutes the largest human ASM survey to date. The results showed methylation states that specifically associated with one allele of a SNP. Strikingly, a major fraction (38-88% depending on the cell line considered) of ASM is determined by CpG dinucleotide-containing SNPs, thus supporting the idea that disruption of CpG sites is a major determinant of differential methylation. This observation is likely to have important implications for genome biology, as databases list >200,000 CpG nucleotide-associated SNPs in the human genome (discussed in [61]). The obvious question is what factors are involved in SNP-operated ASM *cis*-regulation. Results of genomic approaches offer a complex picture, as they suggest that a given CpG dinucleotide-containing SNP impacts DNA methylation if it is included in a region normally targeted by epigenetic regulators [61]. Clearly, these exciting results have to be complemented by mechanistic studies, above all to clarify the role of sequences adjacent to a given SNP as opposed to the effects of the SNP *per se*. A schematic view of phenomena described in this paragraph is presented in Fig. (**1A**).

**Fig. (1):**
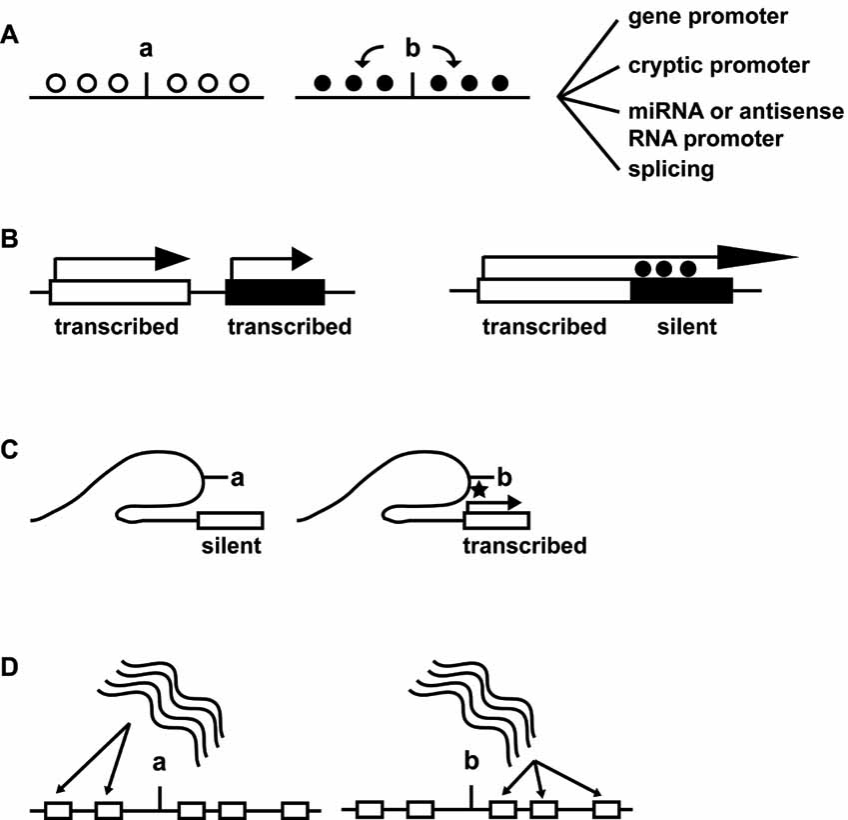
Schematic effects of GV on epigenetic marks. **A**, allele (a and b, respectively)-specific regulation of local DNA methylation states and examples of possible biological effects. Vertical lines mark GV position. Open and closed circles represent unmethylated and methylated residues, respectively. The b allele is hypothetically represented as the one associated with DNA hypermethylation spreading to adjacent sequences (arrows). **B**, normal expression patterns of two adjacent genes (left) are altered if a deletion in polyadenylation signal sequence of the upstream gene causes transcript extension to and silencing of a downstream gene (right). Closed circles on the right indicate hypermethylation of the overrun promoter of the downstream gene. **C**, long-range transcriptional impact of GV-associated epigenetic marks. The b allele is associated with enhancer-like histone marks (star) positioned in a chromatin loop extending to a gene promoter (white rectangle). **D**, noncoding SNP-containing transcripts (curved lines) regulate expression and epigenetic marks of different target genes (white boxes) depending on genotype. See [2,58,59,63,64,72] and text for details.

In addition to a single nucleotide GV, a deletion or insertion can impact epigenetic marks in neighbouring sequences. This phenomenon has been documented in a variety of models, as in the classic example the *A^vy^* allele of the mouse *Agouti* gene. The *A^vy^* allele contains an upstream inserted intra-cisternal A particle (IAP) retrotransposon, which determines the erasure of repressive DNA methylation marks and ectopic Agouti expression [62]. A recent report illustrates the occurrence of a similar mechanism in a gene that is relevant for human health [63]. The authors addressed mechanisms of the DNA mismatch repair *MSH2* gene silencing in cancer-prone Lynch syndrome patients. The intriguing observation was that *MSH2* promoter hypermethylation and silencing was associated with active transcription of a mutated allele of the epithelial cell adhesion molecule gene (*EPCAM* or *TACSTD1*), located upstream to *MSH2.* The mutated *EPCAM* allele bears a 3` end deletion encompassing polyadenylation signals, resulting in transcript extension into the *MSH2* gene. The authors propose that unscheduled transcript extension-induced repressive epigenetic marks may be a common source of gene silencing (Fig. **1B**). This provoking idea is yet to be confirmed, but the notion that unscheduled RNA polymerase transit through a promoter imposes DNA methylation is plausible in the light of evidence that intragenic DNA methylation is a landmark of transcribed genes (discussed below in paragraph “Genetic variants, epigenetic marks and splicing”).

### GVs and Histone Marks

In addition to DNA methylation, histone marks can be modified by SNPs, as a recent study clearly illustrates [64]. The authors analysed a particularly relevant SNP (rs6983267) in the context of this review, located intergenically and notably distal (335 kb) from the nearest gene, *i.e.* *c-MYC*. The interest in this particular SNP originated from previous genetic evidence that it is significantly linked to risk for a variety of cancer types, and from the fact that mechanisms by which this SNP impacts *c-MYC* transcription remained elusive [65]. Results revealed that rs6983267 is part of a chromatin loop that allows large-distance interaction with the *c-MYC* promoter. Interestingly, the chromatin loop appears to be a constitutive feature of that region, as its presence is not associated to rs6983267 genotype. Rather, the rs6983267 G allele displays an enhancer-like histone mark that accounts for allele-specific *c-MYC* promoter activation [64]. These findings are important, as they show that two chromatin architecture-related mechanisms, *i.e.* a change in histone mark and a facilitating chromatin loop, concur to allow long-range transcriptional control by an intergenic SNP. Therefore, the study provides a model mechanism that can be easily tested for other intergenic or non-coding SNPs (Fig. **1C**).

### Genetic Variants, Epigenetic Marks and Splicing

If the transcriptional impact of GVs that affect epigenetic marks at promoter or enhancers is relatively straightforward to envision, the effects of gene body – *i.e.* introns and exons - chromatin modifications are only beginning to be understood. The first comprehensive study addressing this issue was conducted in the plant *A. thaliana* and revealed that gene body methylation is a feature associated with intermediately expressed genes, rather than being a silencing mark [66]. A recent study provided insights into gene body DNA methylation in humans [67]. The authors mapped genome-wide DNA methylation patterns at progressive differentiation stages between human embryonic stem cells and fully differentiated fibroblasts. A consistent feature of transcribed genes was promoter hypomethylation as expected and, in addition, high gene body methylation. Although these marks correlated with expression, transcriptional effects of gene body methylation may be subtle, as exons were more methylated than introns and a sudden transition in DNA methylation levels marked exon-intron boundaries. The latter observation is important, as it suggests that gene body DNA methylation could be involved in differential splicing, possibly independently of absolute transcript levels. In addition to DNA methylation, histone marks are believed to be involved in splicing, as shown by a study addressing the mechanisms for neural cell adhesion molecule 1 (*NCAM1*) gene exon 18 exclusion upon depolarization of neuronal cells [68]. In this controlled system, depolarization resulted in the accumulation of hyperacetylated histone 3 Lys 9 at the differentially spliced portion of the gene. Interestingly, a slow RNA polymerase II mutant favoured exon 18 inclusion, suggesting that histone marks affect splicing by dictating the elongation rate. A recent review extensively discusses this crucial topic [69]. 

The implication of these findings is that exploring the interplay between epigenetic marks, splicing and protein function may help to interpret cases of inconclusive genetic associations. To mention just a few examples among the ones discussed in the first section of this review, gene body GVs in *MR* and *LEPR* may result in alternative splicing as a result of epigenetic marks that may be under the dual control of GV genotype and environmental factors. Notably, *LEPR* is present in 3 splice variants (NM_002303_._3, NM_001003679.1 and NM_001003680.1, respectively), offering the testable hypothesis that gene body polymorphisms affect DNA methylation states that spread to the alternatively spliced sites.

### SNPs and Non-Coding RNAs

A further layer of complexity in the interplay between GVs and epigenetic marks is represented by micro RNAs (miRNAs). miRNAs are short, endogenous 18-25-nucleotide long RNAs that were originally identified as translational repressors mediating the degradation of specific target mRNAs [70]. miRNAs are of potential interest in the context of this review, as they have been recognized as modifiers of DNA methylation at specific target sequences in plants and therefore possibly in humans as well [71]. Two reports indicate that SNPs can impact on miRNA function. The first shows that a SNP is in principle sufficient to alter maturation of miRNA precursors into shorter active miRNAs [72]. The mechanisms underlying these phenomena are not clear, as SNPs have been detected in miRNA residues involved in target recognition rather than precursor processing. Similar conclusions were drawn by subsequent reports that have been recently reviewed in detail [73]. The second, analysed the impact of intergenic SNPs on miRNA profiles [2]. As we mentioned above, the same study concluded that intergenic SNPs are a significant proportion of human disease-associated SNPs. This information was coupled with histone signature data suggesting that many of these intergenic SNPs may be actively transcribed. The authors went on showing that a number of intergenic SNPs associated with inflammatory and autoimmune diseases are included in 100-200 nucleotide-long transcripts. These intergenic transcripts (referred to in the study as transRNAs) exert dramatic effects on the expression of large numbers of miRNAs and mRNAs. Furthermore, expression of short 52 nn-long sense and antisense SNP-including intergenic transcripts resulted in markedly different, allele-specific miRNA and mRNA signatures involved in a number of disease-related pathways (Fig. **1D**) [2]. Interestingly, specific SNP-containing intergenic transcript alleles regulated a number of chromatin modifier genes, thus directly linking SNPs and epigenetic regulation. 

## CONCLUSIONS

Recent literature offers abundant proof of principle that the interplay between GV and epigenetic marks may be a widespread mechanism of transcriptional regulation. From the point of view of genetic association studies, epigenetics may explain the relevance of non-coding and intergenic GVs. Additionally, epigenetics may shed light on cases of biologically relevant GVs that show contrasting results between different countries and ethnic groups. For example, the interplay between rs6983267 genotype and *c-MYC* activation discussed above is controversial, as a number of studies failed to detect any significant association (discussed in [74]). In particular, the latter study identified a strong association between a splicing variant of the rs6983267-interacting TCF7L2 transcription factor, but not rs6983267 genotype, and *c-MYC* expression [74]. This inconsistency may have multiple explanations, but it may be revelatory of the inherent difficulties in relating epigenetic modifications, GV genotype and phenotype. A scenario can be envisioned, in which diet and environment affect epigenetic marks that control *TCF7L2* alternative splicing and rs6983267 chromatin structure. This view is consistent with the idea that sequences undergoing epigenetic mark transitions, such as some of CpG SNP-containing ones, are expected to be relatively more prone to resetting [51]. It is possible that diet and environment induce epigenetic marks with opposite effects on *c-MYC* transcription, for example locking rs6983267 chromatin in a non-enhancer architecture, but favouring *TCF7L2* splicing variants that activate c*-MYC* transcription. If that were the case, particular population-specific dietary and environmental conditions would mask the association of *c-MYC* expression with rs6983267 genotype to the advantage of its association with *TCF7L2* transcript variants. A simplified view of this process is proposed in Fig. (**2**). From the point of view of type 2 diabetes, whether intronic *TCF7L2* SNPs as the ones discussed in the first section affect local DNA methylation states and consequently splicing in a diet- and environment-dependent fashion, it is to our knowledge a yet unanswered question. 

The evidence discussed in this review suggests that the rich tapestry of genotype-phenotype interactions can in some cases be interpreted only by interdisciplinary genetic association studies that take into account biological features such as transcript structure and level, DNA methylation states, histone marks and high-order chromatin structure, and stratify study populations accordingly. If this assumption holds true, any viable claim to implement personalized medicine will have to deal with those additional components as a complement to individual patient genome sequence information.

## Figures and Tables

**Fig. (2):**
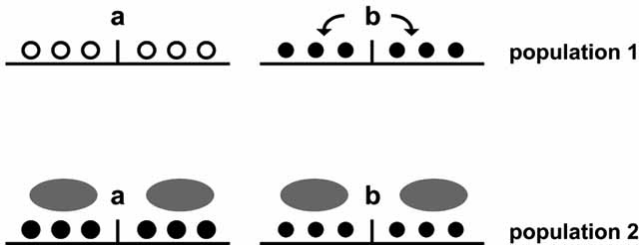
Simplified view of how dietary, environmental and possibly other exogenous factors can interfere with the establishment of allele-specific DNA methylation states and complicate comparisons between genetic association studies conducted in different populations. In population 1, those exogenous factors are weak or neutral and the b allele associates with local DNA hypermethylation compared to allele a (symbols are as in legend of Fig. **1**). In population 2, exogenous factors (grey ovals) distinct in dose or type from the ones affecting population 1 override allele b-specific effects and lock both a and b alleles in a hypermethylated state. If b allele-associated and exogenous factor-induced epigenetic marks are comparable, genetic associations will be masked in population 2. For simplicity, DNA hypermethylation is represented as the effect of diet-related or environmental factors, but DNA hypomethylation is an equally likely outcome.

## ABBREVIATIONS

5mdC: = 5-methyldeoxycytidine
ASM: = Allele-specific DNA methylation
BMI: = Body mass index
GV: = Genetic variant
miRNA: = micro RNA
nn: = Nucleotides
SNP: = Single nucleotide polymorphism
